# Using beat-to-beat heart signals for age-independent biometric verification

**DOI:** 10.1038/s41598-023-42841-4

**Published:** 2023-10-07

**Authors:** Moran Davoodi, Adam Soker, Joachim A. Behar, Yael Yaniv

**Affiliations:** 1 Biomedical Engineering Faculty, Technion-IIT, Haifa, Israel; 2 Laboratory of Bioenergetic and Bioelectric Systems, Faculty of Biomedical Engineering, Technion—IIT, Haifa, Israel

**Keywords:** Biomarkers, Cardiology, Medical research

## Abstract

Use of non-stationary physiological signals for biometric verification, reduces the ability to forge. Such signals should be simple to acquire with inexpensive equipment. The beat-to-beat information embedded within the time intervals between consecutive heart beats is a non-stationary physiological signal; its potential for biometric verification has not been studied. This work introduces a biometric verification method termed “CompaRR”. Heartbeat was extracted from longitudinal recordings from 30 mice ranging from 6 to 24 months of age (equivalent to ~ 20–75 human years). Fifty heartbeats, which is close to resting human heartbeats in a minute, were sufficient for the verification task, achieving a minimal equal error rate of 0.21. When trained on 6-month-old mice and tested on unseen mice up to 18-months of age (equivalent to ~ 50 human years), no significant change in the verification performance was noted. Finally, when the model was trained on data from drug-treated mice, verification was still possible.

## Introduction

The last few decades have witnessed an increase in the use of biometrics for security identification, forensic science and surveillance purposes. Biometrics is based on unique human characteristics, including biological, physical and even behavioral, to identify and verify individuals. Various biometric technologies are available, such as fingerprint, hand-or-face geometry, hand-written signature and voice analysis^1^. However, the relative ease with which such signals can be forged (i.e., circumvention) has limited their biometric reliability^2,3^

Biometric tasks can be classified as identification and verification. The first step in both tasks is enrollment^4^. In this phase, each individual is scanned by some sensor and a digital signature is created. This digital signature is also referred to as a biometric sample or a biometric agent. The identification task is a multiclass classification, in which a biometric algorithm compares the biometric agent (e.g., fingerprints or face images) of a subject who needs to be identified with all other biometric agents in a database of biometric agents. A decision is made as to which of them the subject’s biometric agent matches using a comparison score^2^. It should also have the ability to deny access if no match is found in the database. In contrast, in verification, the decision made by the algorithm is binary: true or false. The comparison is performed only with stored data from a single individual who the subject claims to be. The binary decision is still based on a threshold of a comparison score. Overall, the identification process is a multiclass classification whereas the verification task is a binary classification. Based on the method, an update of the biometric signatures in both tasks is needed.

In the past decade, electrocardiogram (ECG), which registers electrical activity of the heart, has been shown to be suitable for personal identification^5–10^. The reported ECG-based biometric methods are based on 12-lead ECG^5–10^. However, the 12-lead system is not user-friendly and usually requires medical personnel to set up. Although approaches to shift toward single-lead acquisition have been developed^11,12^, a pair of electrodes is still needed. Two electrodes may be cost affordable, but the subject still must be in physical contact with the device for biometric recognition. The heart beat-to-beat time intervals may reflect the uniqueness of the heart’s physiological system and can be measured by compact wearable devices such as watches or wristbands and even remotely by video camera^13^. Moreover, it substantially reduces the data dimensionality needed for verification in comparison to ECG.

This work aimed to design a biometric verification method termed “CompaRR” solely based on heartbeat intervals, which are the time intervals between consecutive R peaks in the ECG signal. Of note, any device that provides information on beat interval can theoretically be used. Practically, Charlton et al.^14^ showed equivalent R peak extraction performance using PPG vs. ECG.

In real-life biometrics, once a subject claims to be person “A”, the algorithm would track the biometric signature of “A” in a database of heartbeat-interval biometric signatures and use a neural network to compare it to the newly measured biometric signature. Before using the neural network for the first time, the database must be acquired and used to train the network. After the training phase, the neural network should be able to compare and make decisions for both subjects’ biometric signatures already existed in the database and for new subjects added to the database, without the need for retraining. Moreover, as biometric signature is only seldom calibrated, it must be confirmed that age has a negligible effect on its performance. Finally, due to the broad use of medications around the world, specifically medications that affect the heart rate (such as beta blockers and ivabradine), the effect of drugs on the performance of the verification task must be examined.

To address all the above challenges, mouse data were used. The mouse lifespan is much shorter relative to humans, which simplifies examination of aging effects on biometric verification performance. A dataset consisting of repeated longitudinal recordings from 30 aging mice (out of 58 in total) from ages 6–24 months was used. All of the 30 mice lived at least 24 months. Moen et al.^15^ calculated the Kaplan–Meier survival curve^16^ on the total 58 mice and found that 50% did not reach the age of 24 months. Thus, the recordings used here are equivalent to ~ 20 to 75 human years since 75 is approximately the current median life expectancy in the western world^17^. Note, that the data are repetitive and such a public database does not exist for humans. Moreover, because mice have a high heart rate relative to humans, a sufficient number of heartbeats can be captured in a short time. Finally, the dataset included heartbeat interval recordings, with and without heart rate-affecting drugs, which can be easily collected in mice.

## Results

### The effect of number of heartbeat intervals on the ability to perform biometric recognition

Two different training processes were used to examine the dependence of biometric verification performance on the number of heartbeats: complete dataset (CD) approach and partial dataset (PD) approach. See “General approach” in “Methods” section.

**Figure 1:**
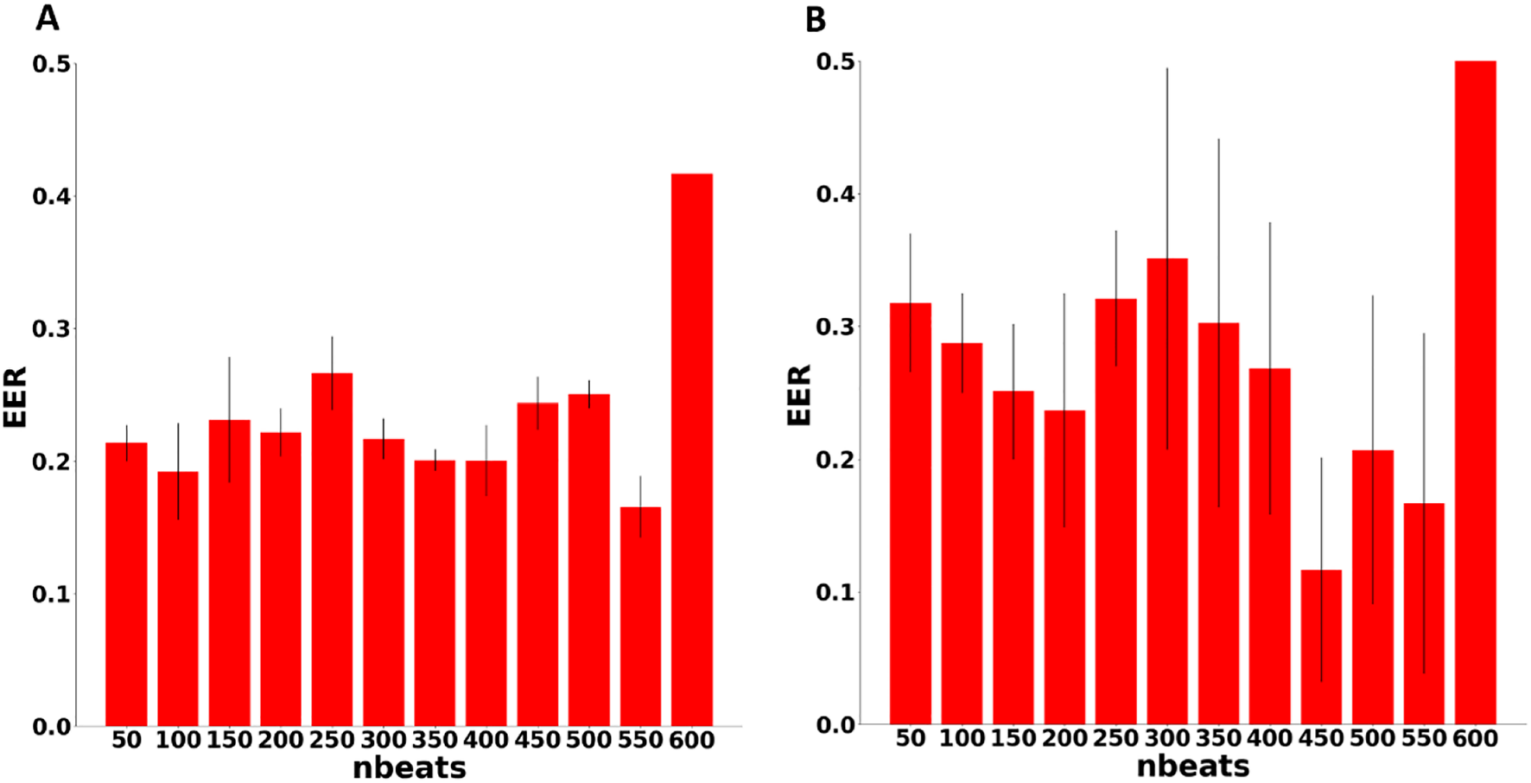
Equal error rate as a function of the heartbeat window length (nbeats). Biometric verification performance on the test set, as measured by equal error rate (EER) at different heartbeat window lengths after the model was trained using the (**A**) complete dataset approach (CD, unseen windows from the same mice) and the (**B**) partial dataset approach (PD, unseen mice).

All training data were from 6-month-old mice and, for this experiment, the tested data were also from mice of the same age. To assess repeatability, the test sets were each split into 5 folds in order to evaluate the mean equal error rate (EER, see definition in the Methods section) and standard deviation, with lower EER indicating better performance. Figure 1 shows the EER for both CD and PD approaches. The minimum EER was 0.17 in the CD experiment and was achieved at 550 beats, and 0.12 in the PD experiment, and achieved at 450 beats. However, the CD coefficient of variation $\left(CV=\frac{\sigma }{\mu }\right)$ at 550 beats was one order of magnitude smaller than for the PD experiment CV at 450 beats. Thus, training with the CD approach is more reliable and less sensitive to data variations, as expected. For other performance measures see Table 1.

**Table 1:** Summary results (*CD* complete dataset, *PD* partial dataset, *EER* equal error rate, *CV* coefficient of variation, *Std* standard deviation, *Basal* no drugs administration, *Drug* with drug administration, *combined* basal + drug).

CD/PD	Minimal EER	CV at minimum	Overall Std
Basal	0.1653/0.116	0.14/1.37	0.027/0.065
Drug	0.133/0.146	0.103/1.2	0.034/0.097
Combined	0.255/0.318	0.042/2.816	0.016/0.025

### The effect of age on biometric verification performance

**Figure 2:**
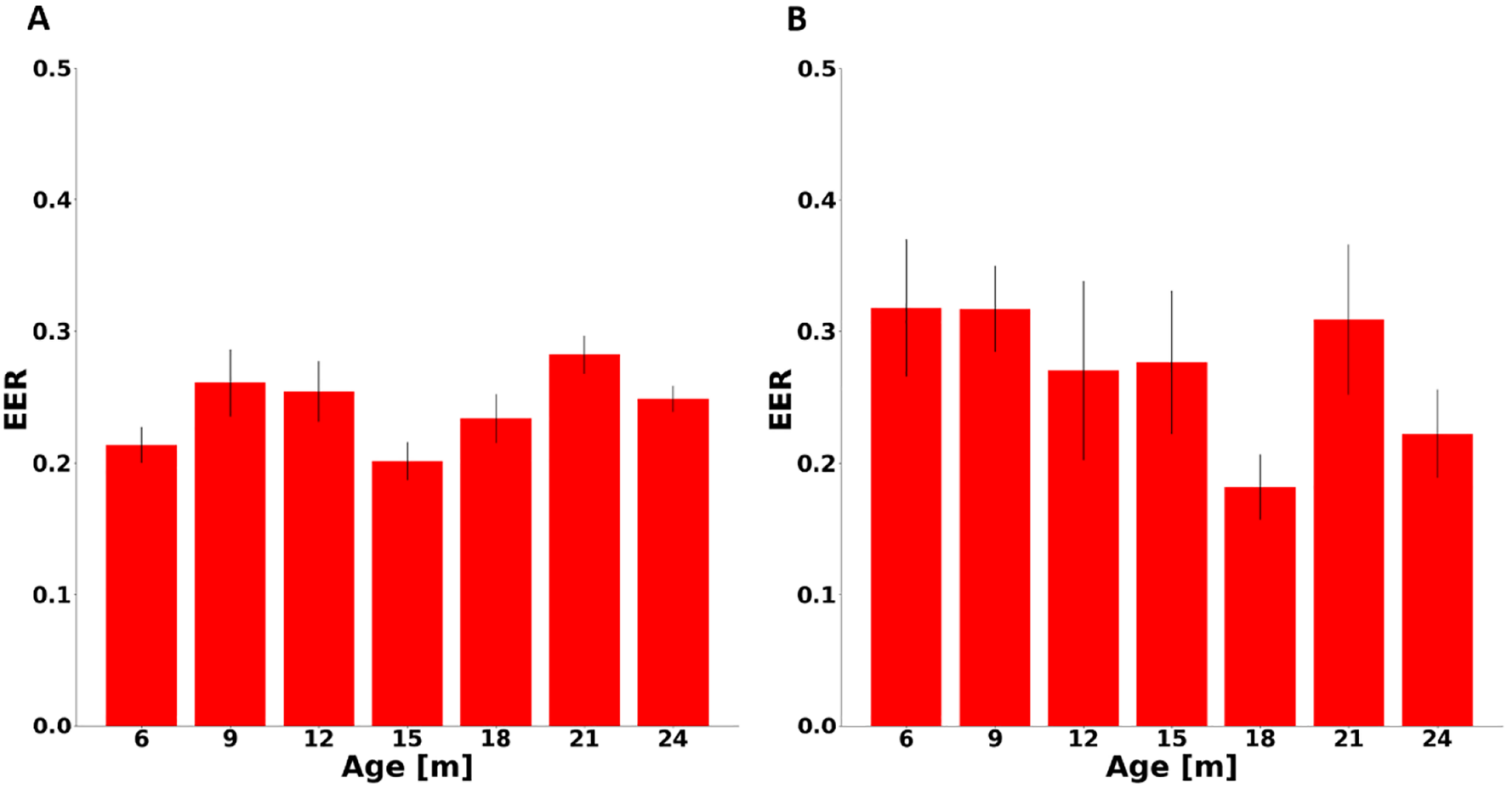
Equal error rate (EER) as a function of age. Biometric verification performance on the test set on data from mice of different ages, as measured by EER after the model was trained using the (**A**) complete dataset (CD, unseen windows from the same mice) approach or the (**B**) partial dataset (PD, unseen mice) approach. The models were trained with heartbeat window pairs collected from mice at the age of 6 months only.

Ideally, the biometric signature is learned once or at least seldom calibrated. Thus, it must be demonstrated that age has a negligible effect on verification performance. To examine the robustness of the proposed method, the model was trained using both the CD and PD approaches, on 50-beat-long heartbeat window pairs from 6-month-old mice and then tested on unseen heartbeat window pairs from mice of older ages. The heartbeat window length of 50 beats was a practically feasible one to acquire (specifically in humans) and provided a sufficient number of heartbeats to allow for good biometric verification performance, (see Fig. 1). Figure 2 shows the EER for both approaches. In the CD-trained model, EER at the age of 15 months was within the equivalence margin of $3\sigma$ of EER at the age of 6 months (see Methods section), with p < 0.05. The p value at the age of 18 was 0.055. In the PD-trained model, data from mice of ages 12 and 15 months were within the equivalence margin defined by the EER at the age of 6 months, with p < 0.05, as were data from mice of ages of 9 and 21 months with p < 0.01. Of note, the standard deviation at the age of 6 months was lower in the CD compared to the PD model (0.013 vs. 0.052) and the overall standard deviation for CD was lower than for PD (0.025 vs. 0.048).

### The effect of drug administration on biometric verification performance

**Figure 3:**
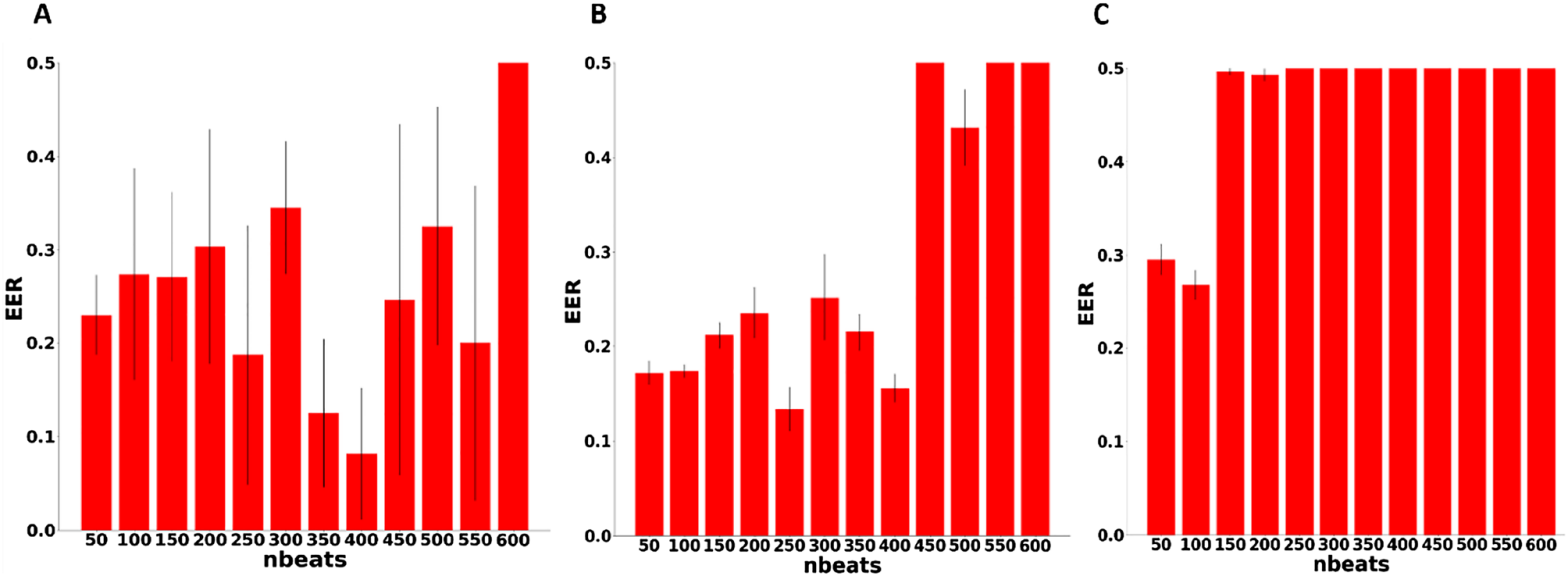
Dependence of equal error rate performance on drug administration measured using a complete dataset (CD) approach. Biometric verification performance on the test set of data collected from 6-month-old mice, as measured by equal error rate (EER) for a model trained on (**A**) heartbeat windows without drug, (**B**) heartbeat windows with drug and (**C**) combined heartbeat windows with and without drug. The test set was identical to all 3 conditions (**A**–**C**) and included heartbeat window with drug data from 6-month-old mice.

Considering the fact that medications are commonly used in everyday situations, the performance of the proposed method was examined on heartbeat windows pairs from mice that had been treated with drugs that affect the heartbeat dynamics by interfering with autonomic nervous system (ANS) input to the heart (such as beta blockers that administered to patients with heart failure). First, the CD approach was used to simulate a condition in which the biometric signature was learned without drugs and the biometric verification was performed in the presence of drugs. Figure 3A shows that the minimum EER of 0.08 was achieved for a heartbeat window of 400 beats. This EER was 50% lower compared to the performance on untreated mice (Fig. 1A; EER: 0.17). However, the CV was now 6 times higher, indicating the instability and high variance among different heartbeat windows. Next, the CD approach was used to simulate conditions under which the biometric signature was learned in the presence of drugs and the biometric recognition was performed in the presence of drugs as well. In this case, a minimum EER of 0.13 was achieved at a heartbeat window length of 250 beats (Fig. 3B). Note that the overall standard deviation (excluding EER = 0.5) was similar to that measured where the model was trained without drugs and performance evaluated without drugs (e.g., Fig. 1A; 0.027 vs. 0.034). Similar outcomes were obtained when using the PD approach (Supplementary materiel, Fig. S1). Finally, the CD approach was applied to simulate conditions in which the biometric signature was learned on data recorded in the same mice before and after drug administration and was tested in the presence of drugs. Each pair was composed from heartbeat windows under the same condition (with drug or without drug). Figure 3C shows that the minimum EER of 0.26 was achieved for the heartbeat window of 25. Under these conditions, the system performance was poor. Similar outcomes were measured when testing the model performance on a combined dataset (with and without the presence of drugs) using the PD approach (Fig. S2).

### The effect of drug administration on the ability to perform biometric verification with increasing age

The robustness of the methods was assessed by training in either approach on heartbeat windows from 6-month-old mice and testing it on heartbeat windows of drug-treated mice of older ages. Window pairs of 50 heartbeats were used for the same reasons mentioned above.

**Figure 4:**
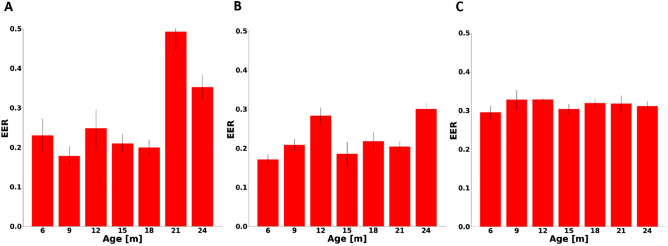
Equal error rate performance as a function of age in the presence of cardiac drugs using complete dataset (CD) approach. Biometric verification performance on a test set, as measured by equal error rate (EER) for a model trained on (**A**) heartbeat window pairs without drug, (**B**) heartbeat window pairs with drug and (**C**) combined heartbeat window pairs with and without drug. The test set was identical to all 3 conditions (**A**–**C**) and included heartbeat window with drug data from 6-month-old mice.

Using CD approach, the biometric signature was learned from data of untreated, 6-month-old mice and the biometric verification was performed on data from treated mice of different ages. Figure 4A shows that a minimum EER of 0.18 was achieved at the age of 9 months. The EERs were within the equivalent margin defined by the EER at the age of 6 months at 9 months (p < 0.05) and at the ages of 12, 15 and 18 months (p < 0.01). Taken together, EER was age-independent until the age of 18 months. Next, using the CD approach, the biometric signature was learned from data of drug-treated 6-month-old mice and the biometric recognition was performed on data from drug-treated mice of different ages. Figure 4B shows that a minimum EER of 0.17 was achieved at the age of 6 months. None of the EERs of the older ages were within the equivalent margin of 6-month EER. Finally, performed using the CD approach, the biometric signature was learned on data from treated and untreated 6-month-old mice and the biometric verification was performed on data from treated mice of older ages. Figure 4C shows that the EER did not change along different ages, but the system performance was poor.

## Discussion

Biometric verification is widely used for security-based applications. However, some biometric methods are either easy to forge or require high-cost devices. This work demonstrated the feasibility of a heartbeat interval-based biometric verification method under real world limitations. The system termed “CompaRR” can perform verification on subjects whether they were part of the training process or entirely new and added to the database after training. Moreover, the method proved aged-independent under certain circumstances. The biometric signature was learned at the age of 6 months (equivalent to 20 human years) and proved accurate for verification until the age of 24 months (equivalent to 75 human years). In addition, the method performed well whether the calibration and test were performed with or without drugs. Therefore, the method can be used as complementary biometric agent for other widely-used biometrics for verification tasks.

As expected, throughout all of the experiments, better performance was achieved when the system was trained with the data from all of the mice and tested on unseen heartbeat windows (i.e., the CD approach). Although both CD and PD can occur in reality, the PD experiment is more complicated because it requires a very high level of generalization and the ability to learn the overall mechanism of comparison. Note that even when the hyperparameters shown in Table S1 were tuned for PD assignment, they still performed better in CD. In both cases, a naïve classifier (EER = 0.5) was obtained at around 600 beats. Ideally, the number of beats should be low so data acquisition and biometric inference can be quick. On the other hand, a unique pattern will be more likely to be discovered in longer signals because dynamics can be measured in long signals. Learning a pattern often requires statistics to be constant or at least not vary so much, i.e. the signals should be at least wide sense stationary (WSS). However, the likelihood of a non-stationary signal increases with larger windows and thus the EER is also likely to increase. Moreover, the number of heartbeat windows for both training and testing decreases with higher numbers of heartbeats which limits capability of generalization and makes the training unstable.

It is well known that heartbeat dynamics change with age^18^ Ideally, a biometric signature should be learned once or at least seldom calibrated. Thus, age should have only a weak effect on identification performance. The model trained on heartbeat windows of data from 6-month-old mice and tested on data from mice of older ages, showed that the biometric signature can be learned once and effectively used when the examined individual ages.

The performance of the proposed method was tested in three different scenarios with data from drug-treated mice (Fig. 3). When the training and testing data were both of heartbeat windows in the presence of drugs (Fig. 3B), the performance in some aspects was similar to the case when learning and testing data contained no drugs (Fig. 1). This included similar minimum EER (achieved at 550 beats in the absence of drugs and 250 beats in the presence of drugs, p < 0.05) and CV values at the minima (0.14 vs. 0.103) and overall standard deviation (0.027 vs. 0.34). This suggests that as long as the training and testing were performed under the same conditions (with or without drug), performance will be good. Additional evidence supporting the importance of the given condition is provided in Fig. 3A, where the performance was reduced when the signature was learned using data obtained without any drug but evaluated on heartbeat windows in the presence of drug.

The methods were verified on mouse data. Note, that the mice were genetically related and therefore the performance achieved is expected to be lower than for unrelated individuals. The unique dataset enabled testing of the effect of aging and drugs on biometric verification, which to date, has been inadequately tested. Mice experiments are commonly used to study and model human physiology, specifically in modeling cardiac diseases^19^. Zaragoza et al.^19^ concluded that mice model is always be possible to devise an appropriate clinical strategy for human, and thus, animal models remain the best tools for understanding the mechanism of human cardiovascular diseases. In addition, Han-Feng and Jin^20^ showed that by lowering the heart rate of a mouse to be at the intermedium range of human, heart rate to be used as reliable model for humans. Finally, Kaese and Verheule^21^ showed that a scaling needs to be done to humans but in general, mice cardiovascular models can mimic the electrophysiology of the human heart. Future translation to the clinic will require acquisition of heartbeat intervals dataset. Note that, ideally, the data should be recorded in repetitive measures and not as one continuous measure that was segmented. Considering the stationarity of the heartbeat interval signal, a one-minute scale of human heartbeats should be used^22–24^, regardless the different number of heartbeats in mice.

The presented results mouse identity can be verified using only heartbeat window pairs and a simple Siamese network composed only from convolutional and linear layer. Table S2 shows that the number of examples was quite low and it possible that more data will improve our results. According to Ingale et al.^25^ the EER measure achieved by ECG-based methods tested on human was superior to the results presented here.

An acceptable EER in biometric verification depends both on the method used (i.e., face recognition, fingerprint, voice recognition etc.) and the number of individuals, but mostly ranges between 0.1 and 10%^3^. Ingale et al. compared almost 40 different ECG-based algorithms, with different or overlapping datasets, with reported EERs ranging between 0.2 and 19.15% for sets with the number of subjects ranging from 6 to 1019. However, it seems that most of the reported EERs were determined on validation sets and not on proper test sets. Moreover, none of the works used the PD approach, and there was no evaluation on unseen human without retraining. In addition, their comparison showed the sensitivity of performance to different phases such as preprocessing, feature extraction techniques and segmentation from the entire ECG signals which are much more complicated phases comparing to RR signals. Note that many of the reported algorithms first excluded many noisy ECG recordings to improve performance and all used long recordings that were segmented, which is not ideal.

Our present method holds the advantage of the ability to extract a signal without a costly ECG device which is also complicated to connect. Heartbeat windows can be extracted with many devices, including smart watches and photoplethysmogram (PPG) and even remotely by video camera. Another important benefit is the substantial dimensionality reduction achieved using this method. For example, a heartbeat window with fixed 250 beats has an average duration of 33 s in mice. In comparison, the ECG used for peak detection was sampled at 10 kHz. Thus, an equivalent (in duration terms) ECG window would have on average 335,000 samples. With a beat window containing only 250 samples (beats), dimensionality was reduced by 1340 folds.

### Limitation

Heart rate variability depends on the activity state, e.g., running, sleeping^26^, and on psychological state, e.g., stress and sadness^27,28^. Our recordings were taken from mice under anesthesia. To compare to other activity states along the day, 24 h ECG halters of five awake 3-month-old mice^29^ were tested with our model trained without drugs. As before, we extracted the peaks and filtered the RR signals using the PhysioZoo platform^30^. The trained model was run with different numbers of beats (50–600) and the performance along the day was examined. The performance was poor. When only 10 min along the day from each mouse were used, we were able to achieve a minimal EER of 0.31 and a mean value of 0.46. It is possible that different states, different phases in the circadian rhythm and the fact that the mice were awake were the main cause of these poor performances. In addition, the anesthesia itself is a drug and its effect on biometric performance cannot be isolated. The PD approach was of course the approach we had to take with the new unseen mice. Additional data that the model will train on in a CD approach may improve the results.

## Methods

### General approach

The main goal of this work was to provide a proof of concept that biometric verification can be performed from heartbeat-interval time series only. In biometric tasks, verification involves comparison of a pair of input signals and should estimate whether the signals comprising the pair belong to the same subject or not. In our case, the pair is composed simply of two heartbeat windows, i.e. two RR time series that contain the same number of beats. The windows can either belong to the same mouse, just from different time periods in the recording, or belong to two different mice. In order to accomplish this goal, two different training processes were used. In the complete dataset (CD) approach, a neural network was trained with heartbeat window pairs from the data of all subjects and the performance was evaluated on unseen heartbeat window pairs. In this manner, the CD approach simulates verification on a subject that was a part of the database used for training. In the partial dataset (PD) approach, the model was trained on the entire heartbeat window pairs of some mice and tested on heartbeat window pairs of unseen mice. This approach simulates the scenario per which a previously unseen individual is to be verified based on an existing set of biometrics of other individuals, without retraining or without adding information to the database.

The robustness of our method was examined by testing its performance with data from subjects at different ages. Ideally, biometric signature is learned once or at least seldom calibrated, meaning that age should have only a weak influence on performance. Thus, the algorithm was always trained on data from 6-month-old mice (see below) and tested on data from mice of older ages. Robustness to age was tested using a two-sided equivalence test (TOST) approach. Unlike classical hypothesis testing, equivalence tests are used to validate the fact that a difference between two expected values is within a given margin. The null hypothesis states that the two expected values are different, i.e., do not fit inside the margin and, thus, rejection means that they do fall within the margin. For this task, we performed two one-sided t-tests (TOST^31^) using the “scipy” package^32^ and examined whether the sum of the two p values was lower than the given significance level $\alpha$. The specific test selected was the unpaired T-test with non-equal variances, with equivalence deviation (margin) of 3 times the standard deviation $\left(3\sigma \right)$ of EER at the age of 6 months.

Since the relationship between heartbeat interval patterns and biometric signature of an individual are unknown, neural network was used. Out of the known architectures, the most suitable one for verification task is the one that measurers similarity as a Siamese neural network does. A Siamese neural network is composed of two identical neural networks, each of which receives an input, sends it to the same latent space and there makes the comparison^33–35^. This seems to perfectly suit our verification task, as it is a binary comparison task. Direct comparison between two RR inputs seems impossible; therefore, the comparison was made in the latent space specifically designed for this purpose by choosing our loss function.

### Dataset

The original dataset was comprised of 58 C57/BL6 mouse ECG recordings and their extracted heartbeat signals published in Moen et al.^15^ The recordings were collected in accordance with the Guide for the Care and Use of Laboratory 102 Animals published by the National Institutes of Health (NIH Publication no. 85–23, revised 1996). Experimental protocols were approved by the Animal Care and Use Committee of the National Institutes of Health (protocol #441-LCS-2016).

Mice were anesthetized with 2% isoflurane (Baxter Corp), administered at rate of 0.2 ml/min, and electrode needles were inserted under the skin. Data were analyzed from the beginning of the recording (at least 20 min after anesthesia). ECG signals were recorded using Power Lab 6, at a sampling rate of 10 kHz. Three-lead electrocardiograms were recorded at constant temperature (25 °C) and humidity (44%) for 10 min under basal conditions (i.e., no drugs) and 40 min after injection of a saline solution (400 µL for 30 g body weight) containing atropine (75 µg/mL) and propranolol (150 µg/mL), denoted as drugs. Only one lead data was used for analysis. During recordings, a heat lamp was positioned at a constant distance (approximately 25 cm) from the mouse’s body to prevent heat loss. Starting at 6 months of age, ECG time-series were recorded at 3-month intervals until the age of 24 months, to evaluate verification performance as a function of age. Mice who lived for less than 24 months were excluded from the database. Overall, from 58 mice, 30 mice were included in the database.

The duration of the basal state was always much shorter than the “drug” state. For a balance compression, the mouse with the smallest number of windows in the basal state defined the number of heartbeat windows, denoted as N. For mice with a larger number of heartbeat windows the first N in both basal and “drug” states was selected. The number of pairs varied with the number of beats, as shown in Table S2.

### Dataset pre-processing

Overall, each ECG signal is subjected to a peak detection process for R peaks detection. Then, the time interval between each peak is calculated. The RR intervals are calculated as $RR\left({t}_{i}\right)={t}_{i+1}-{t}_{i}$, where ${t}_{i}$ are the times where the peaks were detected. ${\left\{RR\left({t}_{i}\right), {t}_{i}\right\}}_{i=1}^{N}$ is called RR time series. Only the heartbeat intervals, i.e. the RR time series, were used as input to the system. Heartbeat intervals were extracted using PhysioZoo^30^. RR time series with N samples (N beats) is referred to as a heartbeat window with a length of 50 beats. The heartbeat windows were non-overlapping. Once all of the RR time series were extracted from the ECG signal, the ECG signal was no longer needed.

The first 2 min of the “drug” segments were excluded to avoid transients. Range-based filtering from PhysioZoo^30^ was used. The filter range was defined as 0.05–0.24 s, corresponding to heart rate ranges of 250–1200 bpm (beats per minute). In this filtering method, the RR intervals which are out of range are ignored and a linear interpolation between the nearest normal beats is made.

### Neural network

The verification model was based on a variation of the Siamese network^36^. The network inputs were heartbeat window pairs, which can either belong to the same mouse and then be considered positive pairs or can belong to different mice and then be considered negative pairs. Each minibatch consisted of 50% positive pairs and 50% negative pairs.

**Figure 5:**
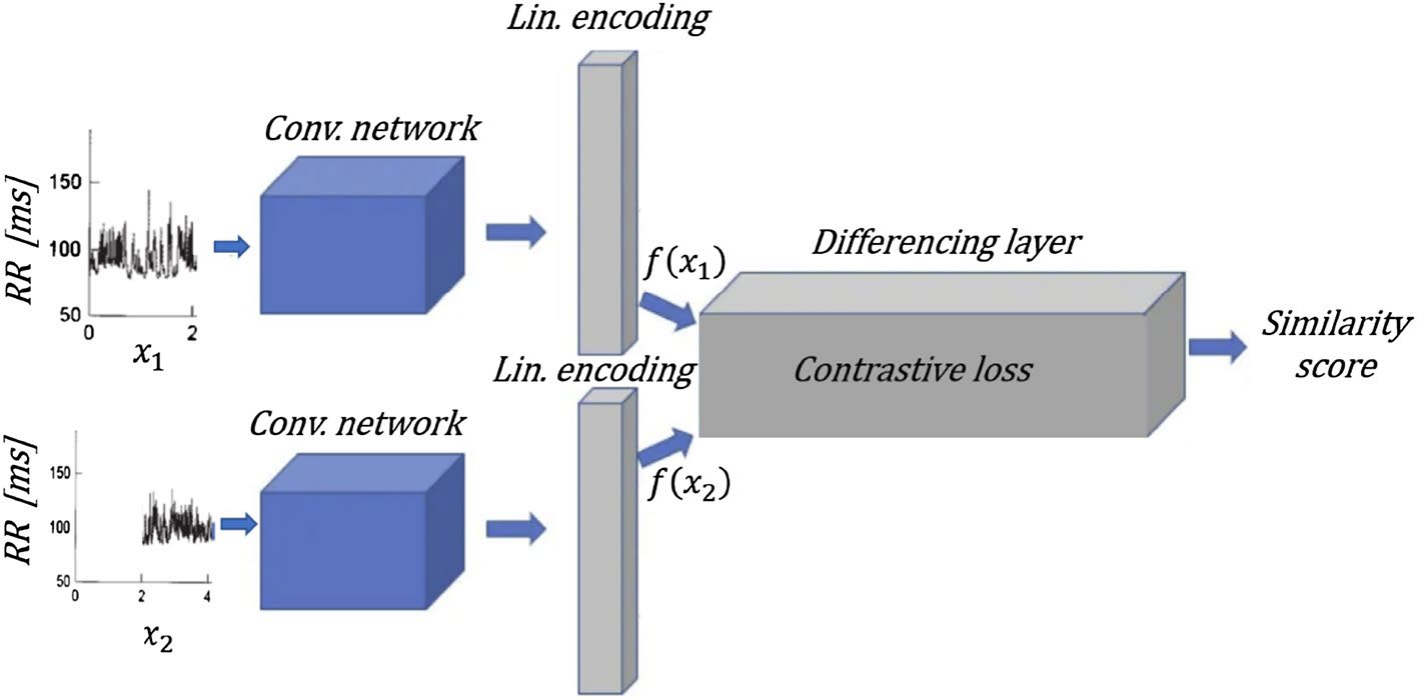
Flow chart Siamese network. The similarity score is based on the cosine similarity function. In this figure an illustration of a positive pair as an input (RR time series taken in different times from the same mouse) is provided. We train the model to have a high similarity score for positive pairs and low similarity score for negative pairs. The full architecture built using “Pytorch”^38^ is detailed in the methods section under “Neural network”. Image was adapted from^39,40^.

In Fig. 5, we show a conceptualize Siamese neural network that receives in its inputs two RR time series from the same mouse (0–2 s and 2–4 s). Thus, this is a positive pair. The encoding layers are the linear layers as detailed below.

The model contained three convolutional layers, followed by batch normalization and MaxPooling (n = 2) and activation by a rectified linear unit (ReLU). These layers had the same kernel size which was equal to $\frac{1}{10}$ of the heartbeat window length. Then, three linear layers were regularized by dropout, followed by batch normalization and activation by ReLU, except the last layer that was only linear. The learning rate and batch size were adjusted to each condition (no drug, drugs or combined) by Bayesian search^37^ and described in Table S1.

### Training process

A modified contrastive loss function that is based on cosine similarity, i.e. the measured angle between the embedded vectors, was introduced. The mean batch loss function over B pairs is as follows:

1 $$L\left(\Theta \right)=\frac{1}{B}\sum_{i=1}^{B}\left(1-{y}^{\left(i\right)}\right)c\left({x}_{1}^{\left(i\right)},{x}_{2}^{\left(i\right)};\Theta \right)-\lambda {y}^{\left(i\right)}\left(b+c\left({x}_{1}^{\left(i\right)},{x}_{2}^{\left(i\right)};\Theta \right)\right)$$

$${y}^{(i)}=\left\{\begin{array}{cc}1& if\,tag\left({x}_{1}^{\left(i\right)}\right)=tag\left({x}_{2}^{\left(i\right)}\right)\\ 0& otherwise\end{array}\right.$$

$$c\left({x}_{1}^{\left(i\right)},{x}_{2}^{\left(i\right)};\Theta \right)=\frac{{f}^{T}\left({x}_{1}^{\left(i\right)};\Theta \right)f\left({x}_{2}^{\left(i\right)};\Theta \right)}{{\Vert f\left({x}_{1}^{\left(i\right)};\Theta \right)\Vert }_{2}{\Vert f\left({x}_{2}^{\left(i\right)};\Theta \right)\Vert }_{2}}=cos\left(\gamma \right),$$

where $tag\left({x}_{1}^{\left(i\right)}\right)=tag\left({x}_{2}^{\left(i\right)}\right)$ means that the first component of pair number $i$ came from the same mouse as the second component. $f$ is the Siamese network parameterized with learnable $\Theta$. $c\left({x}_{1}^{\left(i\right)},{x}_{2}^{\left(i\right)};\Theta \right)$ represents the cosine similarity calculated as the normalized inner product between the two embedded vectors $f\left({x}_{1}^{\left(i\right)};\Theta \right),f\left({x}_{2}^{\left(i\right)};\Theta \right)$ in the latent space. It also represents a measure of the "angle" between them, denoted here as $\gamma$. The terms $\lambda >0$ and $b\in [-\mathrm{1,1}]$ were added to control the tradeoff between false acceptance rate (FAR) and false rejection rate (FRR). $b$ defines whether FAR or FRR is more important and $\lambda$ tells how important that measure is. If $b>0$, then FRR is more important and if $b<0$, FAR is more important. $b$ and $\lambda$ were set as hyperparameters and the optimal ones that would minimize the loss function were sought. A visualization of the loss function with the minimal and maximal values of $b$ can be seen in Fig. S3.

The hyperparameters of the model in each condition (no drugs, drugs or combined) were separately tuned using a Bayesian search^37^. The hyperparameters tuned within the models and written in Table S1 were: batch size, dropout, learning rate, optimizer's momentum and weight decay, number of epochs and $b,\lambda$ [Eq. (1)].

These hyperparameters were tuned according to a heartbeat window length of 250 beats for each condition (no drugs, drugs or combined) separately and applied over all of the heartbeat window lengths, ranging from 25 to 600 beats.

The preparation of the CD experiment included a partition of all of the data from thirty 6-month-old mice into training and testing windows, with a ratio of 80%-20%, respectively. Note, that because the number of heartbeat windows decreases with increase in the heartbeat window length, there were cases of mice with only one single heartbeat window in the testing set. Thus, the positive pair of these specific mice had to contain two identical signals. The percent of such windows was negligible up to heartbeat window length of 600 beats.

For PD experiments, the training, validation and testing set were each comprised of data of different sets of mice. For the 6-month-old data, the training set included 24 mice and the testing set included 6 mice.

### Model evaluation and statistical methods

For each experiment, pairs of heartbeat windows were compared. Negative pairs were defined as 0 and positive as 1. In the confusion matrix, $C\in {\mathbb{N}}^{2\times 2}$, the rows represent the ground truth labels and the columns represent the predictions. To predict whether a pair is positive or negative, a threshold was set for $c\left({x}_{1}^{\left(i\right)},{x}_{2}^{\left(i\right)};\Theta \right),$ which can range between – 1 and 1. This is equivalent to setting a threshold to the angle between the embeddings. If the angle between the embeddings of the pair components is larger in absolute value than the threshold, then the pair would be classified as negative and if the angle is smaller, the pair would be classified as positive. The FAR and FRR are a function of the threshold. These measures are equivalent to false negative rate and false positive rate, respectively.

In biometry verification statistics, it is common to report EER^41^ which corresponds to the value of FPR and FAR when they are equal.

**Figure 6:**
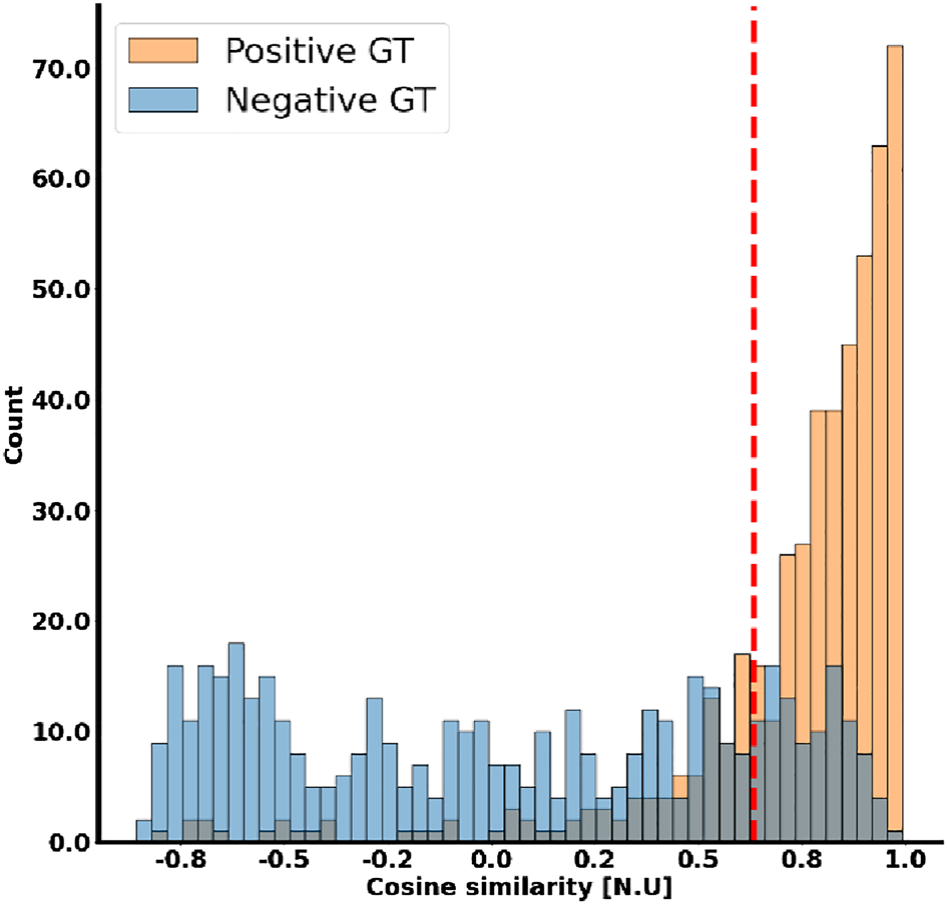
Visualization of the classification task. The decision boundary is shown as a red dashed line. The orange and blue colors are for ground true labelling of pairs. A perfect classifier would have only orange examples to the right of the threshold and only blue examples to the left of it.

Wellness of performance can be visualized as shown in Fig. 6, where the drug model was tested with a window length of 25 heartbeat. The cosine distance between each pair of the embeddings in the latent space was measured. A negative pair should tend to be closer to − 1, whereas a positive pair should tend to be closer to + 1. The decision boundary is a threshold in the range of [− 1, + 1].

### Supplementary Information

Supplementary Information.

## Data Availability

The data that support the findings of this study are available from NIA but restrictions apply to the availability of these data, which were used under license for the current study, and so are not publicly available. Data are however available from the authors upon reasonable request (Yael Yaniv, yaely@bm.technion.ac.il) and with permission of NIA/NIH.
