# Supplementary material for: Using beat-to-beat heart signals for age-independent biometric verification
Source: Sci Rep. 2023 Oct 7;13:16937. doi: 10.1038/s41598-023-42841-4 (PMC10560207; doi:10.1038/s41598-023-42841-4)
Supplement: Supplementary file 1 — Supplementary Information. [file 41598_2023_42841_MOESM1_ESM.docx]

**Using beat-to-beat heart signals for age-independent biometric verification**

**Supplement**

**Davoodi et al. Beat-to-beat biometric verification**

Moran Davoodi^1^, Adam Soker^1^, Joachim A. Behar^1^ and Yael Yaniv^1*^

^1^ Biomedical Engineering Faculty, Technion-IIT, Haifa, Israel

**^*^Correspondence:**

Yael Yaniv, PhD
Laboratory of Bioenergetic and Bioelectric Systems, Faculty of Biomedical Engineering, Technion—IIT, Haifa

Email : [yaely@bm.technion.ac.il](mailto:yaely@bm.technion.ac.il)
Phone : 972-4-8294124
Fax : 972-4-8294599

# Supplementary material

######
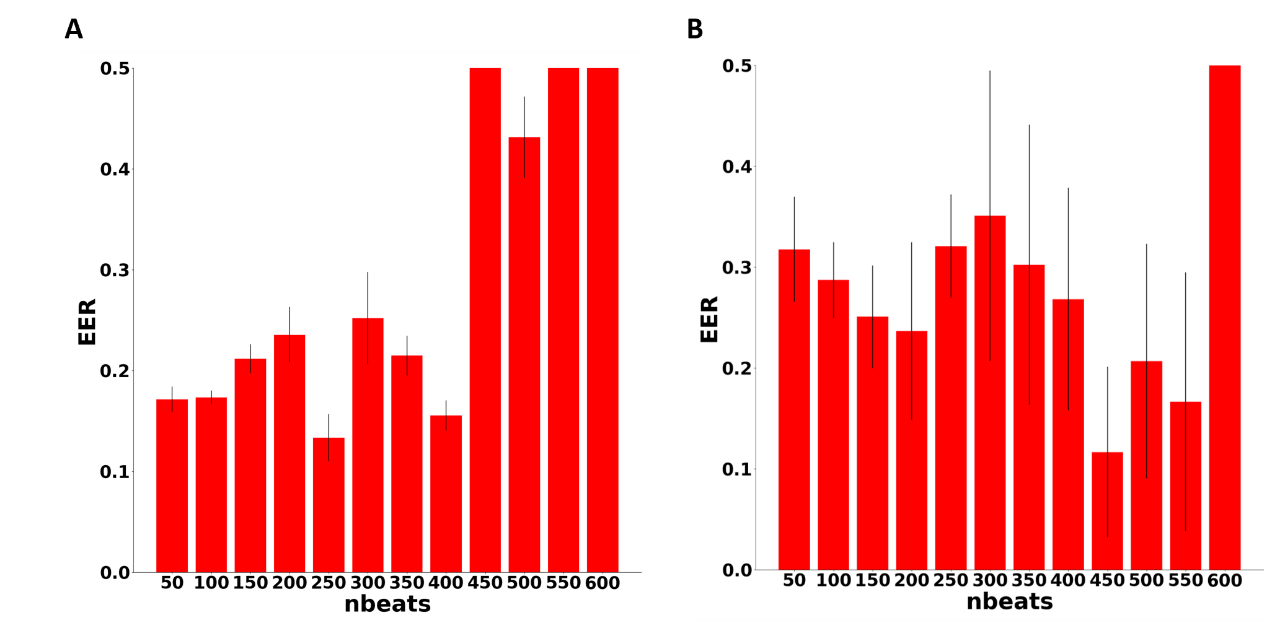


**Figure S1: Equal error rate as a function of the heartbeat window length in the presence of drugs**. Biometric verification performance on a test set, as measured by equal error rate (EER), of the (A) complete dataset approach (CD) used for training and the (B) partial dataset (PD) approach used for training at different heartbeat window lengths, in the presence of drugs.


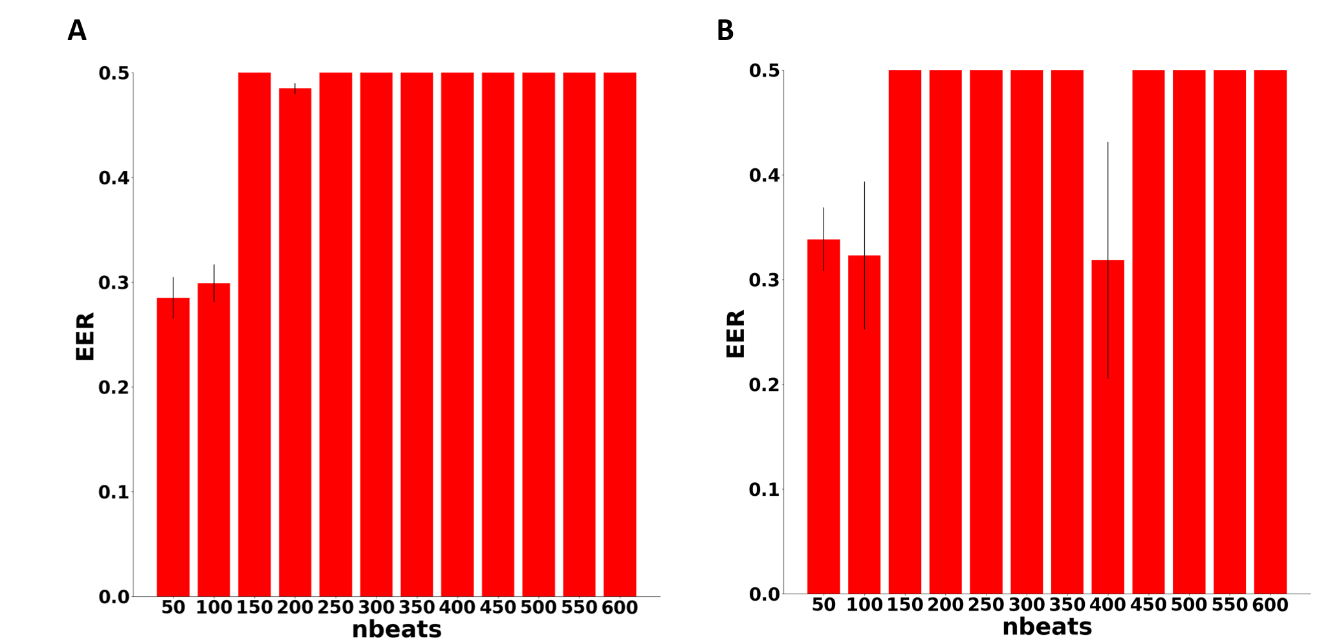


**Figure S2: Equal error rate as function of the heartbeat window length when trained and tested on data in the presence and absence of drugs.** Biometric verification performance on the test set, as measured by equal error rate (EER), for the (A) complete dataset approach (CD) used for training and the (B) partial dataset (PD) approach used for training at different heartbeat window lengths.


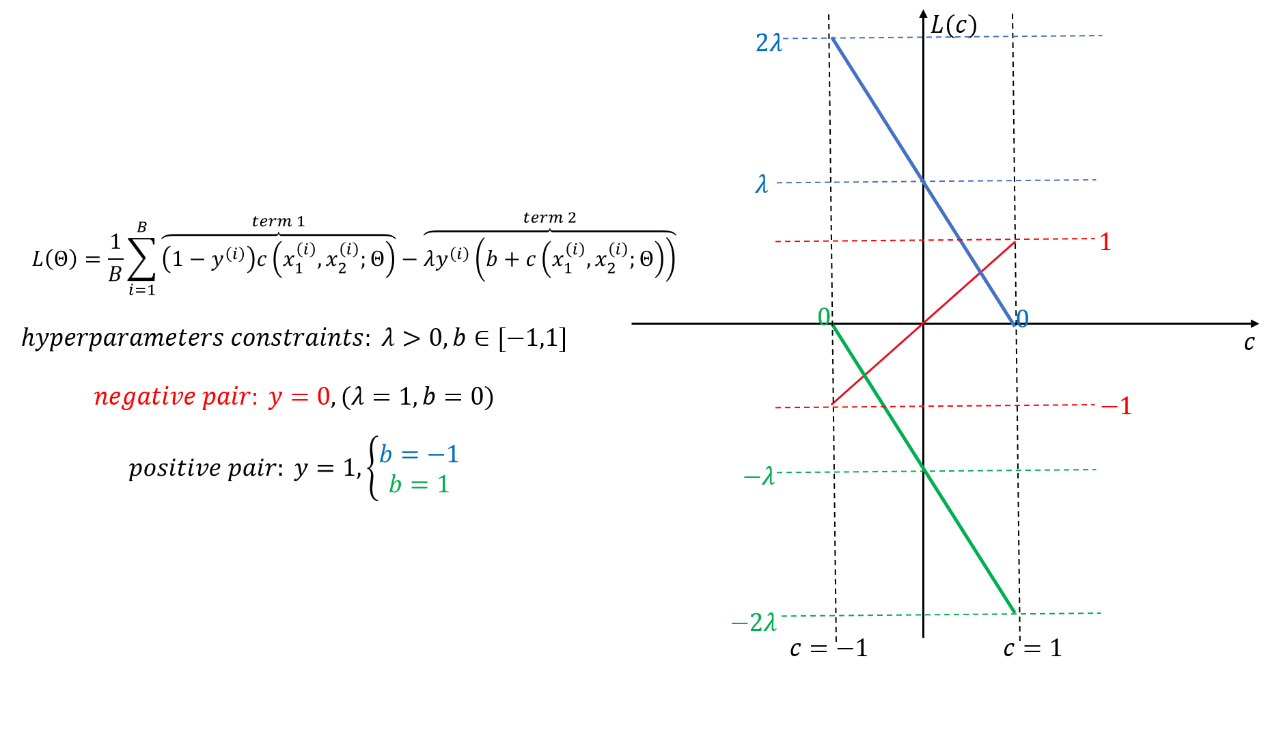


**Figure. S3. Schematic description of the loss functio**n. Loss is composed of two terms. A term is activated according to the label of the pair. If the pair is negative, the loss function activated on this pair is the red one. If the pair is positive, a linear monotonic decreasing function is activated. The linear function is parallel to and in between the blue and green linear functions, depending on the value of $b$.

Tables:

**Table S1. Hyperparameter tuning**

|  | Batch size | Dropout | Learning rate | Momentum | Weight decay | Epochs | $b$ | $\lambda$ |
| --- | --- | --- | --- | --- | --- | --- | --- | --- |
| No drug | 8 | 0.12 | 3.4e-7 | 0.75 | 1.17 | 200 | 0.47 | 9.61 |
| Drug | 16 | 0.34 | 2.7e-7 | 0.7 | 9.8 | 80 | -0.6 | 0.16 |
| Combined | 128 | 0.31 | 4.1e-7 | 0.11 | 0.17 | 319 | 0.48 | 1.31 |

# Table. S2 train/test number of examples

|  | Number of pairs (Nbeats=25) | Number of pairs (Nbeats=600) | Number of windows per mouse (Nbeats=25) | Number of windows per mouse (Nbeats=600) |
| --- | --- | --- | --- | --- |
| Drug or No Drug | 3888/972 | 144/36 | 127/25 | 5/1 |
| Combined | 7776/1944 | 288/72 | 254/50 | 10/2 |
